# A feasibility study of an integrated couples‐based supportive programme for Chinese couples living with colorectal cancer

**DOI:** 10.1002/nop2.700

**Published:** 2020-11-26

**Authors:** Xingjuan Luo, Jieyu Li, Qian Cao, Liya Sun, Ying Chen, Jie Zhao, Qiuping Li

**Affiliations:** ^1^ Wuxi Medical School Jiangnan University Wuxi China; ^2^ Affiliated Hospital Jiangnan University Wuxi China

**Keywords:** blended intervention, cancer, colorectal cancer, couples‐based intervention, oncology, spousal caregiver

## Abstract

**Aim:**

To test the feasibility, acceptability and preliminary efficacy of using an integrated approach combined online and in‐person delivery to support colorectal cancer (CRC) patients and their spousal caregivers coping positively with cancer together.

**Design:**

A single‐arm pre–post‐feasibility design was used.

**Methods:**

Chinese CRC patient‐partner dyads (*N* = 24) accessed the blended intervention combined online platform and face‐to‐face sessions for six weeks between October 2019 to January 2020. Feasibility was measured through recruitment and retention and acceptability was examined by intervention engagement and post‐treatment programme evaluation. Effect sizes were calculated using the complete data (*N* = 20 couple dyads) to evaluate preliminary treatment effect.

**Results:**

Recruitment (70.6%) and retention rates (83.3%) supported programme feasibility. Participants’ positive intervention engagements and evaluations indicated acceptability. The overall small‐to‐medium improvements in most outcome measures verified preliminary efficacy of the integrated couples‐based supportive programme. The findings support its feasible and acceptable for couples coping with CRC and show potential efficacy.

## 1 INTRODUCTION

As one of the most prevalent cancers worldwide, colorectal cancer (CRC) ranks third and second in terms of incidence and mortality, with incidence and mortality rates varying widely in different regions (Bray et al., 2018). According to cancer statistics, the burden of CRC is expected to increase in China in the near future, with nearly 642,300 new CRC cases and 221,100 deaths estimated to occur in 2025 (Zhang et al., 2019).

Cancer diagnosis and treatment affect both patients and spousal caregivers, leading to a growing recognition of couples‐based interventions (Badr & Krebs, 2013; Regan et al., 2012). Reviews have reported that couples‐based interventions had small‐to‐medium beneficial effects on psychological distress, communication, relationship and quality of life (QOL) (Badr & Krebs, 2013; Regan et al., 2012). Our previous “Caring for Couples Coping with Cancer (4Cs)” programme, a single group/uncontrolled study delivered via traditional in‐person dyadic sessions, also showed promising effects on Chinese couples dealing with mixed cancer (Li et al., 2015).

## 2 BACKGROUND

In the context of CRC, evidence has shown that both CRC patients and their partners are affected during the cancer journey (Traa et al., 2015a) and associations and an interdependent nature exist between CRC patients and their partners in several areas, for example, fatigue (Traa et al., 2016), sexual function and marital function (Traa et al., 2015b). A study also reported that relationship quality within patient‐partner dyads affected a couple's adjustment to CRC, specifically, good relationship functioning benefits psychosocial adjustment (Kayser et al., 2018).

The complex mutual impacts between couples resulted from cancer also existed among Chinese CRC patients and their spousal caregivers. Recently, we conducted a qualitative study and found that CRC presents various challenges for Chinese couples and mutual support played a significant role in couples’ journey coping with CRC together (Li et al., 2018). Although a telephone‐based intimacy enhancement intervention targeting American CRC couples has shown promise (Barsky Reese et al., 2014), it only centred on addressing sexual concerns of couples, rather than focusing on a comprehensive dyadic level, for example dyadic mediator, dyadic appraisal and dyadic coping to help CRC couples coping with cancer. No specific interventions focusing on couples coping with CRC on a dyadic level in China were identified. Given the critical need described above, based on the previous 4Cs programme, to satisfy Chinses CRC couples’ unmet needs (Li et al., 2018), a new 4Cs: CRC dyadic programme was developed, with the intention of better supporting CRC couples coping with cancer on multiple dyadic levels.

Web‐based intervention delivery showed unique advantages over in‐person interventions, including diverse format and content, fewer space‐time restrictions and anonymity (Luo et al., 2020). However, possible disadvantages included a lack of personal interaction(Luo et al., 2020). To best make use of the advantages of both the Internet and traditional delivery formats, we combined an online platform with face‐to‐face sessions into one integrated programme, in a blended intervention. This study aimed to examine the feasibility, acceptability and preliminary efficacy of the 4Cs: CRC programme for CRC patients and their spousal caregivers.

## 3 METHODS

### 3.1 Study design and participants

This was a pre–post‐single‐arm intervention study design targeting Chinese CRC patients and spousal caregivers. Eligibility criteria were as follows: adult married couples with one partner diagnosed with CRC (any stage); the patient was cared for by his/her spouse; couples had daily access to a smartphone; and both patient and partner could communicate in Mandarin and were willing to participate in the programme. Participants were recruited in the oncology wards of a cancer hospital in Wuxi City, China, from October 2019 to January 2020. Basic CRC couple demographic and health‐related information was collected pre‐intervention (Table S1).

### 3.2 Guiding theory

**FIGURE 1:**
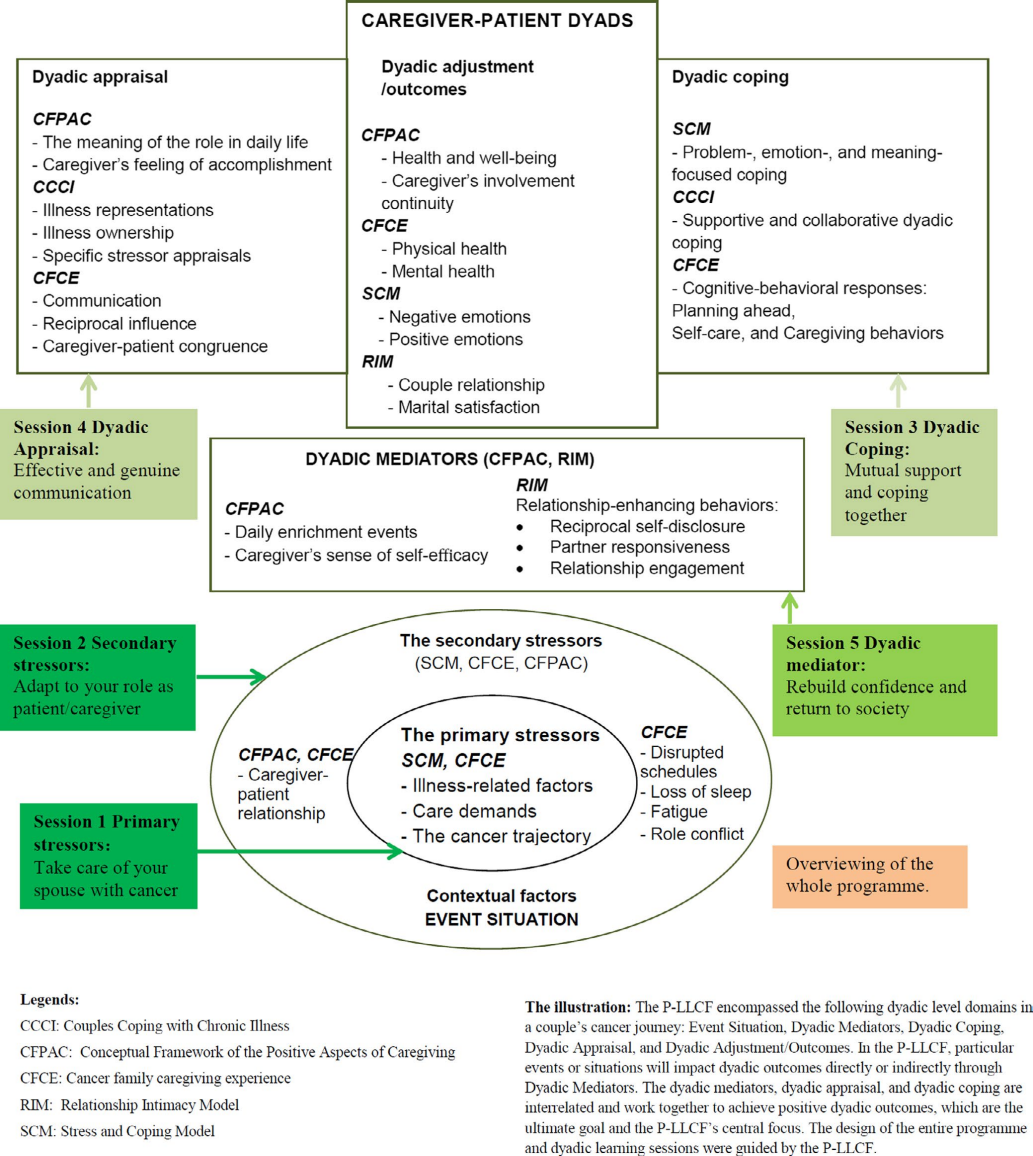
A preliminary Live with Love Conceptual Framework (P‐LLCF) for Cancer Couple Dyads

The design of the dyadic intervention was guided by a preliminary Live with Love Conceptual Framework (P‐LLCF) (Figure 1), which was specifically focused on patients‐partners coping with cancer as a unit during the cancer period(Li & Loke, 2015). The P‐LLCF encompassed the following dyadic level domains in a couple's cancer journey: Event Situation, Dyadic Mediators, Dyadic Coping, Dyadic Appraisal and Dyadic Adjustment/Outcomes. In the P‐LLCF, particular events or situations will have an impact on dyadic outcomes directly or indirectly through Dyadic Mediators, which situated above the action wheel, act as ‘‘leverage’’ to balance or offset the stressors, leading to the dyadic appraisal, coping and adjustment of the cancer couple dyads. The dyadic mediators, dyadic appraisal and dyadic coping are interrelated and work together to achieve positive dyadic outcomes, which are the goal and the P‐LLCF’s central focus. These domains were the foundation for the entire intervention design and dyadic learning sessions.

### 3.3 Intervention development and content

To satisfy CRC couples’ unmet information needs (Li et al., 2018) and because translating the dyadic CRC psychoeducation sessions into a web‐based format could increase intervention accessibility, an online platform (4Cs: CRC programme) was developed, following the recommendations proposed by a review of dyadic web‐based interventions (Luo et al., 2020) and providing information support, psychoeducation sessions, online communication and skills‐building to support couples coping with CRC. The development of the 4Cs: CRC programme was guided by the P‐LLCF (Li & Loke, 2015).

The online platform consisted of six modules: Dyadic Learning Sessions, Health Information, Cancer News, Online Support, Sharing Circle and Personal Centre. Dyadic Learning Sessions was the central module and included five psychoeducational sessions: Take care of your spouse with cancer; Adapt to your role as patient/caregiver; Mutual support and coping together; Effective and genuine communication; and Rebuild confidence and return to society.

### 3.4 Measures

Feasibility was evaluated by calculating recruitment and retention rates. Acceptability was determined by face‐to‐face session completion rates, online intervention engagement and postintervention programme evaluation. Multiple instruments were used to measure CRC couples’ outcome domains pre‐ and postintervention (Table S2). All measures were demonstrated to be reliable in the previous 4Cs programme (Li et al., 2015). Considering the “leverage” effect of Dyadic Mediators in the P‐LLCF (Figure 1), self‐efficacy (dyadic mediator) was identified as a primary outcome, measured by the 12‐item Cancer Behavior Inventory (CBI‐B), which evaluates self‐efficacy in people coping with cancer. The CBI‐B Cronbach's α coefficient ranged from 0.84 to 0.88 (Heitzmann et al., 2011).

Secondary outcome measures consisted of the 37‐item Dyadic Coping Inventory (dyadic coping), 15‐item Cancer‐Related Communication Problems within Couples Scale (dyadic appraisal) and four measures for dyadic outcomes, including the medical outcomes study 12‐item short form (QOL), 14‐item hospital anxiety and depression scale (negative emotions), 17‐item benefit‐finding scale (positive emotions) and 14‐item revised dyadic adjustments scale (marital satisfaction). Other measures included a basic demographic and health‐related information sheet (pre‐intervention) and postintervention programme evaluation questionnaire (Table S2).

### 3.5 Ethics and procedures

All study procedures were approved by the Jiangnan University research ethics committee (JNU20200312IRB09). Once eligible couples gave written informed consent, the dyads independently completed baseline survey measures. They were instructed on how to access the online platform and create a login account prior to study commencement. During the six‐week period, the programme was delivered in a combined format. Weekly reminders were sent to participants asking them to complete each dyadic session. Three couples‐based biweekly (two‐, four‐ and six‐week, respectively) face‐to‐face sessions (each 60–90 min) were held to revisit the online learning sessions and provide additional support if required. The postintervention assessment was administered immediately after intervention completion (at six weeks).

### 3.6 Data Analyses

Descriptive analyses were used to measure feasibility and acceptability. Due to the small sample size, which limited our power to make inferential statistics as to study variables, we calculated the effect size for patients and their partners separately (using mean pre–post‐change scores/pooled standard deviation). The effect sizes were estimated using Cohen's *d*, and the effect was classified as large (*d* = 0.8), medium (*d* = 0.5) and small (*d* ≤ 0.2), respectively (Portney & Watkins, 2009). Participants’ pre–post‐intervention improvement was also assessed using minimally clinically important differences (MCID). Analyses were conducted using SPSS version 25.0.

## 4 RESULTS

**FIGURE 2:**
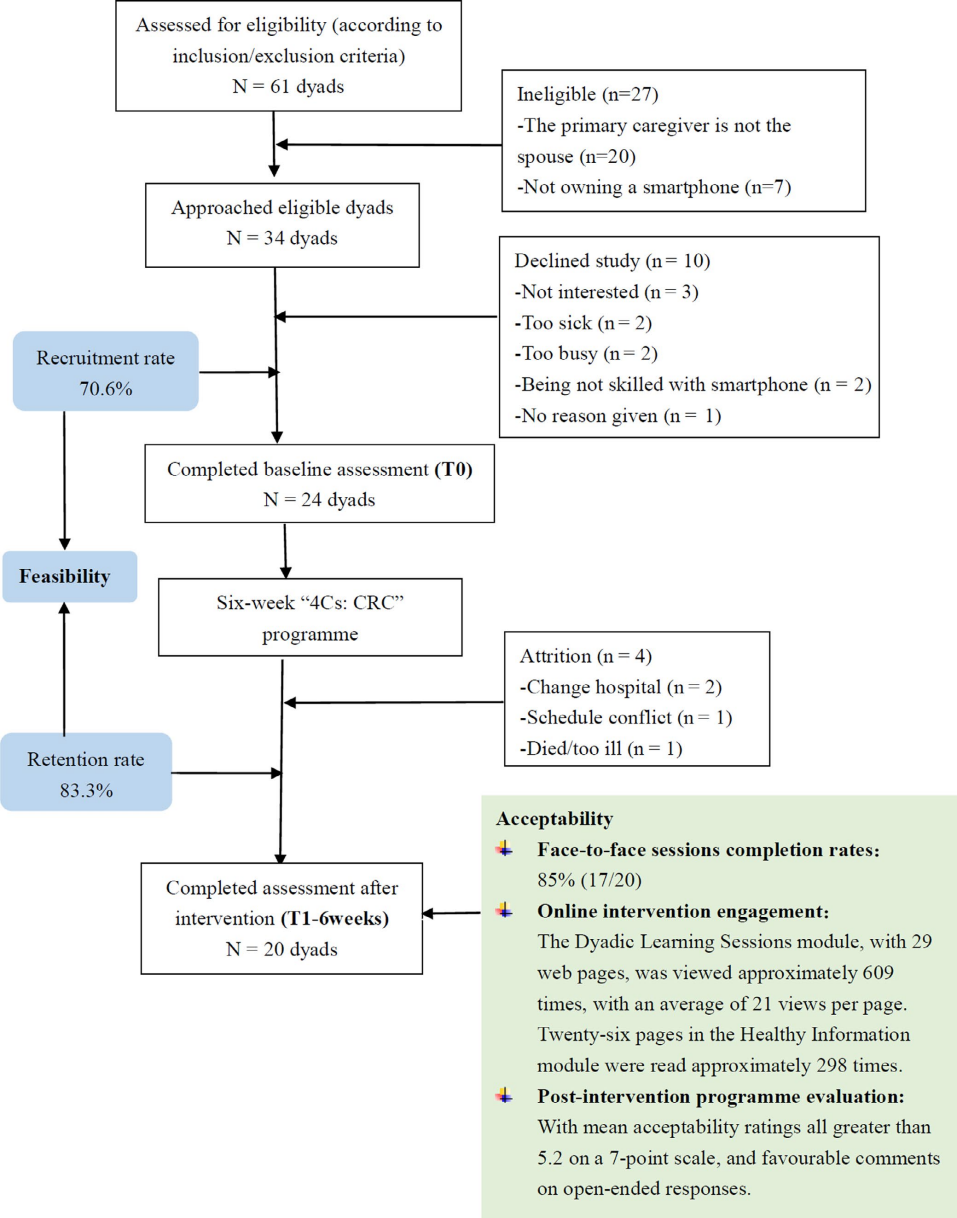
Study consort diagram

Figure 2 illustrates the flow of participants into the study, indicating 70.6% and 83.3% for the recruitment and retention rates, respectively. In terms of face‐to‐face dyadic session engagement, there was an 85% completion rate. Regarding online intervention engagement acceptability, with total approximately 609 views (mean = 21 views per page) for the dyadic learning session module by all included dyads. And the mean view times of each session's pages by per dyad ranged from 3 to 7 views. In addition, participants rated the programme highly in terms of its usefulness, ease of use, as well as satisfaction, with all mean acceptability ratings greater than 5.2 on a 7‐point scale. Open‐ended responses were generally favourable in the postintervention programme evaluation (Table 1).

**TABLE 1:** Feasibility and acceptability outcomes

Feasibility	
Recruitment rates	70.6% consented and enrolled
Retention rates	83.3% completed both pre‐post study assessments
Acceptability	
Face‐to‐face sessions completion rates	Of the 20 dyads that completed post‐intervention assessment, 17 dyads completed all three sessions, yielding an 85% completion rate.
Online intervention engagement	The Dyadic Learning Sessions module, with 29 web pages, was viewed approximately 609 times, with an average of 21 views per page. Twenty‐six pages in the Healthy Information module were read approximately 298 times.
Post‐intervention programme evaluation questionnaire**^†^**	With mean acceptability ratings all greater than 5.2 on a 7‐point scale and favourable comments (see Table 1a and b, below, for detailed information)

1a: Post‐intervention acceptability items among patients and their spousal caregivers (*n* = 20 patient‐spousal dyads)		
Category	Patient *M* (*SD*)	Spousal caregiver *M* (*SD*)
Usefulness		
The programme content is useful	5.5 (1.2)	5.8 (0.8)
The online platform is convenient for me and saves me time when I use it	5.2 (1.0)	5.4 (0.8)
The online platform includes important information that I want	6.0 (0.7)	5.9 (0.7)
Ease of use		
The online platform is easy to use	5.6 (0.9)	5.8 (0.8)
I found what I was looking for quickly and easily	5.5 (0.8)	5.7 (0.5)
The online content is easy to understand and follow	5.6 (0.9)	5.6 (0.7)
Satisfaction		
I am satisfied with the intervention content	5.3 (0.6)	5.7 (0.6)
I am satisfied with the delivery format (combination of online and in‐person delivery)	5.4 (0.7)	5.6 (0.7)
I would recommend it to someone else	5.6 (0.6)	6.0 (0.6)
The programme has		
Increased my knowledge of colorectal cancer	6.0 (0.7)	6.0 (0.7)
Improved my ability to cope with cancer with my partner together	5.7 (0.8)	6.1 (0.8)
**1b: Detailed responses to the open‐ended question**		
There were a total of 11 CRC patients and 12 spousal caregivers who responded to the question. Most of the responses were positive (11/11 and 11/12 respectively). Their favourable comments and suggestions for further improvement of the programme are summarised below:		
*Favourable comments:* Compared to seeking information from the Internet, the online platform provided credible information whenever we wanted it. The information was very practical and easy to understand, e.g. the video on changing an ostomy bag. The content was novel, in providing communication and supportive techniques for couples to work together, topics that had often previously been ignored. It’s really good with a combined format. In fact, I prefer to look through the information online, but my partner is gregarious and outgoing and likes in‐person discussions better, so we can help each other and work as a team.		
*Suggestions for improvement:* Updating information more frequently Providing more detailed information about diet and nutrition targeting different treatment stages (e.g., before and after surgery) might be helpful. It was fatiguing to read too much text, with preference for a presentation with more pictures and less text.		

Note: Postintervention programme evaluation questionnaire**^†^**: the questionnaire was adapted from the existing USE scale (AM. L. Measuring usability with the USE questionnaire. Usability Interface. 2001;8(2):3–6). It evaluated the programme in terms of usefulness, ease of use, and satisfaction on a 7‐point Likert scale ranging from “1” (“Strongly disagree”) to “7” (“Strongly agree”). In addition, an open‐ended question was added to collect any thoughts or feelings regarding use of the programme or intervention improvement suggestions.

Further programme refinement: The present report supported the programme's feasibility and acceptability, but some limitations remain, and should be addressed in future research. Apart from further refining the related content according to participant suggestions, for example updating information more frequently, and preparing more pictures or videos for greater ease of use, we may try to improve the study design, for example using RCT, recruiting adequate numbers of participants and extending the follow‐up period.

Abbreviations: *M*, mean; *SD*, standard deviation.

In terms of primary outcomes, small‐to‐medium improvements in self‐efficacy were found in CRC patients (*d* = 0.36) and spousal caregivers alike (*d* = 0.37). Additionally, improvements were found in self‐efficacy, with 55% of patients and 60% of partners showing a clinically important difference postintervention. Overall small‐to‐medium improvements were also found across all other outcome measures for CRC patients (*d* = 0.12–0.65) and spousal caregivers (*d* = 0.004–0.37) (Table S3). Approximately 30%‐55% of patients and 20%–60% of spouses reported clinically important differences across other outcome measures postintervention (Table S4).

## 5 DISCUSSION

The findings largely supported the feasibility of the hybrid approach. The recruitment rate (70.6%) was excellent, higher than typical rates for most cancer couples‐based interventions, but somewhat lower than that of the 4Cs programme (86.7%). Retention and completion rates were good (83.3% and 85%, respectively), slightly higher than the 4Cs programme retention rate (78.6%) (Li et al., 2015), which could be attributed to the shortened number of face‐to‐face sessions (three vs. six) and the attraction of the online intervention (e.g. flexible and diverse presentation form). Nevertheless, the somewhat lower recruitment rate and higher retention rate of the 4Cs: CRC programme may indicate the potential of blended delivery to enhance intervention adherence, supporting the programme's feasibility and acceptability. This may also point to a need for more publicity, to increase the recruitment rate.

Further, patients and their partners praised the programme for its usefulness and ease of use. Their comments also indicated that the programme delivery mode might be acceptable, but cannot be generalized to a controlled trial, since this is a single‐arm design, and all participants received the study intervention.

The intervention's preliminary effect showed generally small‐to‐medium effect sizes for CRC patients and their spousal caregivers in multiple dyadic domains, similar to our prior 4Cs programme(Li et al., 2015). However, preliminary efficacy is necessarily viewed with caution due to the small sample size.

Although testing in a larger‐scale study is warranted, our study findings might indicate that integrated intervention as a novel delivery approach offers the following potential advantages. First, it combines the advantages of online and in‐person delivery and requires less in‐person contact than single face‐to‐face sessions, making more comprehensive, accessible, minimally intensive psychological interventions possible. Second, the comprehensive strategy adjusts to the unique preferences of each couple, which may to a certain extent enhance intervention adherence.

### 5.1 Limitations

First, this study lacked a control group, so we cannot conclusively attribute the improved results to the intervention. In addition, the small sample size limits the potential to make inferences about study variables. The pre–post‐study design, with no follow‐up, restricts exploration of the long‐term programme's efficacy. Finally, the study was implemented in China and its findings may not be generalizable to a broader population.

### 5.2 Implications for practice

Our findings not only encourage other researchers to best integrate the advantages of the Internet and traditional delivery to support CRC couples, but also to faciliate clinicians in transforming the 4Cs: CRC programme into standard clinical service for CRC couples, allowing them to better adjust to living with cancer.

## 6 CONCLUSION

The 4Cs: CRC programme is a unique, important and promising new approach that appears to be feasible, acceptable and preliminarily effective among Chinese CRC couples. Following participants’ suggestions for improvement (Table 1), testing in a larger‐scale study is warranted.

## CONFLICT OF INTEREST

The authors declare no conflicts of interest.

## Supporting information

Table S1‐S4

## Data Availability

The dataset(s) supporting the conclusions of this article is (are) available and will be provided on request.
