# Supplementary material for: A feasibility study of an integrated couples‐based supportive programme for Chinese couples living with colorectal cancer
Source: Nurs Open. 2020 Nov 26;8(2):920–6. doi: 10.1002/nop2.700 (PMC7877155; doi:10.1002/nop2.700)
Supplement: Supplementary file 1 — Table S1‐S4 [file NOP2-8-920-s001.doc]

**Table S1. Patient and spousal caregiver characteristics**

|  | **Patient** | **Spousal Caregiver** |
| --- | --- | --- |
| **Characteristics** | **n=20** | **n=20** |
| Age, mean (SD)  Range | 56.4(7.6)  40-71 | 56.8(7.3)  39-69 |
| Gender, n (%) |  |  |
| Male | 13(65.0) | 7(35.0) |
| Female | 7(35.0) | 13(65.0) |
| Length of marriage (years), mean (SD)  Range | 31.9(6.8)  16-42 | |
| Education, n (%) |  |  |
| Primary school or less | 10(50.0) | 14(70.0) |
| High school | 7(35.0) | 3(15.0) |
| University or above | 3(15.0) | 3(15.0) |
| Time since diagnosis (months), mean (SD)  Range | 8.3(5.7)  1-24 |  |
| Length of time as an SC (months), n (%)  <6 months  6 ~24 months |  | 9(45.0) 11(55.0) |
| Type of treatment, n (%) |  |  |
| Chemotherapy | 14(70.0) |  |
| Surgery | 3(15.0) |  |
| Others | 3(15.0) |  |
| Stoma, n (%)  Yes  No | 3(15.0)  17(85.0) |  |
| Informed about the disease, n (%)^†^ |  |  |
| Well informed | 10(50.0) | 12(60.0) |
| Partly informed | 10(50.0) | 8(40.0) |
| Health status, n (%) |  |  |
| Feel well：good | 15(75.0) | 12(60.0) |
| As usual | 3(15.0) | 8(40.0) |
| Does not feel well: bad | 2(10.0) | 0(0.0) |

Note: SC: Spousal caregivers；SD: standard deviation

^†^Well informed: The patient fully understood his/her condition; or the SC was well informed about the spouses’ disease.

^†^Partly informed: The patient was informed of the cancer diagnosis, but not about the severity of his/her condition; or SC was partly informed about the spouse’s disease.

**Table S2 Domains in the P-LLCF and correlating programme outcome instruments**

| **Domains in the P-LLCF** | **Outcome variables** | **Description of instruments** |
| --- | --- | --- |
| *Event Situation* | A basic demographic and health-related information sheet | (i) Demographic data: age, gender, length of marriage, religion, level of education, monthly family income, and financial burden of the family due to cancer treatment;  (ii) Clinical data: time since diagnosis, length of time as a spousal caregiver, type of treatment, whether or not the patient has a stoma, the level of the patient/spousal caregiver has been informed of the disease, and health status. |
| *Dyadic Mediators* | Self-Efficacy | The 12-item Cancer Behavior Inventory (CBI-B) is an instrument evaluating self-efficacy in coping with cancer. The CBI-B Cronbach’s α coefficient ranged from 0.84 to 0.88.^1^ |
| *Dyadic Coping* | Dyadic coping strategies | The 37-item Dyadic coping inventory (DCI) was designed to assess how couples cope with stress as a dyad,^2,3^ and the DCI reliability coefficients ranged from 0.50 to 0.92.^4^ |
| *Dyadic Appraisal* | Communication | The 15-item Cancer-Related Communication Problems within Couples Scale (CRCP) was used to assess couples’ cancer-related communication, with higher scores reflecting more communication problems.^5^ The scale demonstrated good internal consistency in both patients and their partners (Cronbach's α = 0.87, 0.81, respectively).^5^ |
| *Dyadic Outcomes* | Physical and mental health | The Medical Outcomes Study 12-item short form (MOS SF-12).^6^ The two components (Physical Component Summary, PCS; and Mental Component Summary, MCS) of the 12-item versions achieved R squares of 0.905 with PCS and 0.938 with MCS of the SF-36 in a cross-validated Medical Outcomes Study. Test-retest (2-week) correlations of 0.89 and 0.76 were observed for the SF-12 PCS and MCS, respectively in the general U.S. population (n = 232).^6^ |
|  | Negative emotions | The 14-item Hospital Anxiety and Depression Scale (HADS) contained Depression and Anxiety subscales, and both subscales were scored from 0 to 21. Higher scores reflected greater anxiety and depression levels.^7^ The HADS Chinese version demonstrated good internal consistency (Cronbach's α≥0.840) among Chinese cancer patient-caregiver dyads.^8^ |
|  | Positive emotions | The 17-item Benefit-Finding Scale (BFS) was specifically designed to measure positive insights arising from the cancer journey and coping with cancer. Higher scores reflected greater benefits perceived from the cancer experience.^9^ The Chinese version of the BFS (BFS-C) consists of three subscales: personal growth, improved relationships, and acceptance. The overall and three subscales of BFS-C in both cancer patients and family caregivers had good internal consistency, with all of the Cronbach’s α ≥0.819.^10^ |
|  | Marital Satisfaction | Couples’ marital satisfaction was assessed using the 14-item Revised Dyadic Adjustment Scale (RDAS).^11,12^ RDAS scores ranged from 0 to 69, with higher scores (≥48) indicating a better marital relationship.^12^ The Cronbach’s α coefficient of the RDAS was 0.90.^11^ |

Abbreviations: CRC: colorectal cancer; P-LLCF: a preliminary Live with Love Conceptual Framework; QOL: quality of life;

**Outcome Assessment Battery References:**

1. Heitzmann CA, Merluzzi TV, Jean-Pierre P, Roscoe JA, Kirsh KL, Passik SD. Assessing self-efficacy for coping with cancer: development and psychometric analysis of the brief version of the Cancer Behavior Inventory (CBI-B). *Psycho-oncology.* 2011;20(3):302-312.

2. Gmelch S, Bodenmann G, Meuwly N, Ledermann T, Steffen-Sozinova O, Striegl KJZFF. Dyadic Coping Inventory (DCI): A questionnaire assessing dyadic coping in couples. 2008;20(2):185-202.

3. Bodenmann GJBH. Dyadic Coping Inventory (DCI). Test manual. 2008.

4. Ledermann T, Bodenmann G, Gagliardi S, et al. Psychometrics of the dyadic coping inventory in three language groups. 2010.

5. Kornblith AB, Regan MM, Kim Y, et al. Cancer-related communication between female patients and male partners scale: A pilot study. *Psycho-oncology.* 2006;15(9):780-794.

6. Ware J, Jr., Kosinski M, Keller SD. A 12-Item Short-Form Health Survey: construction of scales and preliminary tests of reliability and validity. *Medical care.* 1996;34(3):220-233.

7 Zigmond AS, Snaith RP. The hospital anxiety and depression scale. *Acta Psychiatr Scand.* 1983;67(6):361-370.

8. Li Q, Lin Y, Hu C, et al. The Chinese version of hospital anxiety and depression scale: Psychometric properties in Chinese cancer patients and their family caregivers. *European journal of oncology nursing : the official journal of European Oncology Nursing Society.* 2016;25:16-23.

9. Antoni MH, Lehman JM, Kilbourn KM, et al. Cognitive-behavioral stress management intervention decreases the prevalence of depression and enhances benefit finding among women under treatment for early-stage breast cancer. *Health psychology : official journal of the Division of Health Psychology, American Psychological Association.* 2001;20(1):20-32.

10. Li Q, Lin Y, Xu Y, Zhou H, Yang L, Xu Y. Construct validity of the 17-item Benefit Finding Scale in Chinese cancer patients and their family caregivers: a cross-sectional study. *Supportive care in cancer : official journal of the Multinational Association of Supportive Care in Cancer.* 2017;25(8):2387-2397.

11. Busby DM, Christensen C, Crane DR, Larson JHJJoM, Therapy f. A revision of the Dyadic Adjustment Scale for use with distressed and nondistressed couples: Construct hierarchy and multidimensional scales. 1995;21(3):289-308.

12. Crane DR, Middleton KC, Bean RAJAJoFT. Establishing criterion scores for the Kansas marital satisfaction scale and the revised dyadic adjustment scale. 2000;28(1):53-60.

**Table S3** Means, Standard Deviations, and Effect Sizes of Study Variables

|  | **Patients (n=20)** | | |  | **Spousal caregivers (n=20)** | | |
| --- | --- | --- | --- | --- | --- | --- | --- |
| **Outcomes** | **Pre**  **M (SD)** | **Post**  **M (SD)** | **Effect sizes**  **Cohen’s d** |  | **Pre**  **M (SD)** | **Post**  **M (SD)** | **Effect sizes**  **Cohen’s d** |
| ***Dyadic mediators***  Self-efficacy (CBI-B) | 76.5 (13.1) | 80.8 (10.7) | 0.36 |  | 83.2 (8.0) | 85.8 (5.7) | 0.37 |
| ***Dyadic coping***  Dyadic coping inventory (DCI) | 129.3 (11.7) | 132.3 (12.1) | 0.25 |  | 126.0 (14.8) | 128.9 (13.2) | 0.21 |
| ***Dyadic appraisal***  Communication (CRCP) | 6.2 (3.0) | 5.7 (2.5) | -0.18 |  | 7.0 (2.4) | 6.7 (2.5) | -0.12 |
| ***Dyadic outcomes***  Physical and mental health (SF-12)  PCS  MCS | 40.2 (11.6)  42.5 (6.3) | 42.1 (8.6)  43.5 (4.4) | 0.18  0.18 |  | 47.6 (5.2)  38.7 (5.8) | 47.4 (4.0)  41.3 (5.2) | -0.004  0.33 |
| Negative emotions (HADS)  Anxiety  Depression | 6.4 (3.2)  6.5 (4.7) | 6.0 (2.7)  6.0 (3.5) | -0.14  -0.12 |  | 8.5 (4.0)  9.4 (3.3) | 7.9 (3.3)  8.3 (2.6) | -0.16  -0.37 |
| Positive emotions (BFS-C)  Personal growth  Improved relationship  Acceptance  Marital satisfaction (RDAS) | 64.3 (8.0)  33.6 (5.4)  19.6 (2.4)  11.2 (2.0)  51.8 (6.1) | 67.6 (6.2)  35.1 (4.8)  20.2 (2.1)  12.4 (1.7)  52.7 (5.3) | 0.46  0.29  0.27  0.65  0.16 |  | 64.7 (13.5)  33.5 (8.2)  20.3 (3.8)  11.0 (2.6)  51.4 (5.7) | 67.0 (10.7)  34.5 (6.5)  20.9 (3.3)  11.6 (1.8)  52.0 (5.8) | 0.19  0.14  0.17  0.27  0.10 |
| Abbreviations: BFS-C: The Chinese version of the Benefit-Finding Scale; CBI-B: the 12-item Cancer Behavior Inventory (CBI-B); CRC: colorectal cancer; CRCP: cancer related communication problems; HADS: The Hospital Anxiety and Depression Scale; M: Means; MCS: Mental Component Summary; PCS: Physical Component Summary; QLQ-CR29: EORTC colorectal cancer-specific quality of life questionnaire module; QOL: quality of life; RDAS: Revised Dyadic Adjustment Scale; SD: standard deviations; SF-12: The Medical Outcomes Study 12-item short form. | | | | | | | |

**Table S4 The minimal clinically important differences (MCID) calculation and percentage of participants achieving MCID in outcomes**

| **Reported outcomes** | **ES**  **P S** | **R (measure reliability)** | **SD _baseline_**  **P S** | **ES method ^a^**  **P S** | **SEM method ^b^**  **P S** | **Weighted MCID ^c^**  **P S** | **Achieving clinically important differences (pre-to-post intervention)**  **No. (%)**  **P S** |
| --- | --- | --- | --- | --- | --- | --- | --- |
| Self-efficacy (CBI-B) | 0.36 0.37 | 0.85 | 13.1 8.0 | 4.7 2.9 | 5.0 3.0 | 4.8 2.9 | 11(55%) 12(60%) |
| Dyadic coping inventory (DCI) | 0.21 0.25 | 0.85/0.88 (P/S) | 11.7 14.8 | 2.9 3.1 | 4.5 5.1 | 3.7 4.1 | 10(50%) 9(45%) |
| Communication (CRCP) | -0.18 -0.12 | 0.80/0.73 (P/S) | 3.0 2.4 | -0.5 -0.3 | 1.3 1.2 | 0.9 0.7 | 8(40%) 5(25%) |
| Physical and mental health (SF-12)  PCS  MCS | 0.18 -0.004  0.18 0.33 | 0.89  0.84 | 11.6 5.2  6.3 5.8 | 2.0 -0.02  1.1 1.9 | 3.8 1.7  2.5 2.3 | 2.9 0.8  1.8 2.1 | 7(35%) 6(30%)  11(55%) 12(60%) |
| Negative emotions (HADS)  Anxiety  Depression | -0.14 -0.16  -0.12 -0.37 | 0.84 | 3.2 4.0  4.7 3.3 | -0.4 -0.6  -0.5 -1.2 | 1.2 1.6  1.8 1.3 | 0.8 1.1  1.1 1.2 | 10(50%) 4(20%)  7(35%) 7(35%) |
| Positive emotions (BFS-C) | 0.46 0.19 | 0.81 | 8.0 13.5 | 3.6 2.5 | 3.4 5.8 | 3.5 4.1 | 11(55%) 6(30%) |
| Marital satisfaction (RDAS) | 0.16 0.10 | 0.90 | 6.1 5.7 | 0.9 0.5 | 1.9 1.8 | 1.4 1.1 | 6(30%) 9(45%) |

Abbreviations: P: patients; S: spouses; ES: effect size; SD _baseline_: standard deviation of baseline; SEM: standard error of measurement.

Note: As there was no literature reporting the MCID on our outcome measures, we used the distribution-based approach (ES method and SEM method) to calculate the MCID;

ES method ^a^ calculated MCID as ES×SD _baseline_;

SEM method ^b^ calculated MCID as 1×SD _baseline_ × (1-r) ^1/2^, r is the reliability of the measuring instrument;

Weighted MCID ^c^ =50%* ES method ^a^ MCID+50%* SEM method ^a^ MCID
